# Mechanical Properties and Uniaxial Compression Stress—Strain Relation of Recycled Coarse Aggregate Concrete after Carbonation

**DOI:** 10.3390/ma14092215

**Published:** 2021-04-26

**Authors:** Tian-Wen Chen, Jin Wu, Guo-Qing Dong

**Affiliations:** Department of Civil Engineering and Airport Engineering, Nanjing University of Aeronautics and Astronautics, Nanjing 211106, China; chentw2021@163.com (T.-W.C.); donggq@nuaa.edu.cn (G.-Q.D.)

**Keywords:** carbonation, recycled coarse aggregate concrete, replacement ratio, cubic compressive strength, static elastic modulus, stress–strain curve

## Abstract

The application of recycled coarse aggregate (RCA) made from waste concrete to replace natural coarse aggregate (NCA) in concrete structures can essentially reduce the excessive consumption of natural resources and environmental pollution. Similar to normal concrete structures, recycled concrete structures would also suffer from the damage of carbonation, which leads to the deterioration of durability and the reduction of service life. This paper presents the experimental results of the cubic compressive strength, the static elastic modulus and the stress–strain relation of recycled coarse aggregate concrete (RAC) after carbonation. The results show that the cubic compressive strength and the static elastic modulus of carbonated RAC gradually increased with the carbonation depth. The uncarbonated and fully carbonated RAC show smaller static elastic modulus than natural aggregate concrete (NAC). As the carbonation depth increased, the peak stress increased, while the peak strain decreased and the descending part of the curves gradually became steeper. As the content of RCA became larger, the peak stress decreased, while the peak strain increased and the descending part of the curves gradually became steeper. An equation for stress–strain curves of RAC after carbonation was proposed, and it was in good agreement with the test results.

## 1. Introduction

With the acceleration of the modernization process, the demand for concrete in the construction market is increasing. A large amount of natural coarse aggregate (NCA) was produced and then consumed, which leads to an increasing shortage of natural aggregate resources and brings huge challenges to sustainable development [1]. Meanwhile, the construction and demolition of buildings has also brought about more construction waste. Currently, the application of recycled coarse aggregate (RCA) instead of NCA in actual engineering can significantly decrease the amount of NCA [2] and further alleviate the severe shortage of resources, therefore, the engineering application of recycled coarse aggregate concrete (RAC) had received widespread attention and was actively promoted.

The construction industry, which consumes huge natural resources, is not only the main source of CO_2_ emissions, but also suffers from severe durability due to carbonation. It is widely known that carbonation of concrete is essentially a chemical reaction process of Ca(OH)_2_ and C-S-H with carbon dioxide, respectively [3,4]. This will lead to the decrease of the concentration of hydroxide in the pore solution, which means that the pH value is reduced, and induces the increase of chloride ion concentration, further resulting in the corrosion of embedded reinforcements. Immense efforts are being made to reduce the harm of carbonation to concrete by trying different methods. For example, the replacement of ordinary Portland cement by alkali-activated materials may decrease the emission of carbon dioxide from the root [5]; the application of red ceramics could weaken the carbonation effect of carbon dioxide on RCA [6], carbonation pretreatment of RCA could decrease water absorption of cement mortar samples [7], et cetera.

The mechanical performance of RAC after carbonation has always been a hot spot [8]. Carbonation behavior may induce obvious change in the original mechanical properties of concrete structures, as well as decrease the ductility, directly inducing the damage to concrete structures and the reduction of its service life. The existing and future research on the mechanical behavior of carbonated RAC, on the one hand, could provide theoretical basis for the durability design of new recycled concrete structures, and on the other hand, could supply calculation basis for strengthening existing recycled concrete structures.

Compared to NCA, a large amount of original cracks inside RCA due to accumulated damage and old cement mortar, attached to the surface of RCA; the two above-mentioned factors contribute to the larger water absorption capacity of RCA [9]. Additionally, defects involving capillaries, pores and bubbles in the interior of RAC would affect the carbonation process, further generating an impact on the mechanical properties of carbonated RAC [10].

Buyle-Bodin et al. [11] reported that natural aggregate concrete (NAC) exhibits superior carbonation resistance to RAC due to the lower porosity of NAC. Compared to conventional moist curing, Zhan et al. [12] found that CO_2_ curing could dramatically improve the compressive strength of the concrete blocks incorporating RCA in short time. Niu et al. [13] pointed out that compared with uncarbonated concrete, the cubic compressive strength of carbonated RAC with replacement ratios of 50% and 100% increased by 15.41% and 18.03%, respectively. However, Xiao et al. [14] reported that compared with NAC, old cement mortar attached to the surface of RCA results in an increase of cement dosage, causing the RAC to exhibit better carbonation resistance. Xuan et al. [15] found that the carbonation process could significantly improve the physical and mechanical properties of RCA, and it was attributed to the fact that carbonation could enhance the micro-hardness of the cement mortar surrounding the RCA. Comparing scanning electron micro-graphs of carbonated RAC with that of carbonated NAC, Yang et al. [16] found that there were obvious micro-cracks on the interface between RCA and new cement matrix, and the structure was looser in the interface transition zone (ITZ). Silva et al. [17] investigated the influence of various factors on the anti-carbonation performance of RAC, and proposed that recycled concrete structures could reach the expected service life due to the excellent carbonation resistance of RCA.

The concrete structures in service bore large deformation, therefore, the step of evaluating its ultimate load is essential. The stress–strain relationship of recycled concrete structures subject to external load could realistically reflect their mechanical behavior, and a series of meaningful research results have been reported. Suryawanshi et al. [18] carried out the analysis of the constitutive relationship of recycled concrete with various replacement ratios of RCA, the results suggested that compared with NAC, the peak strain of RAC with 100% replacement ratio increased by about 26%, the elastic modulus and compressive strength of RAC decreased by 38% and 11%, respectively. On the basis of comparing constitutive relationships of RAC at 0%, 50% and 100% replacement ratio, D’Alessandro et al. [19] found that the stiffness of RAC decreased with the increase of low strength RCA content. Xiao et al. [20] gained constitutive relationships of RAC under different replacement ratios (0%, 30%, 50%, 70% and 100%), and proposed the stress–strain models of RAC subject to uniaxial compression load. The results show that the proposed function model of the constitutive relationship of RAC has a high correlation with the experimental results. Based on the experimental stress–strain curves of RAC with different replacement ratios, a calculation model which could highlight the influence of replacement ratios on the stress–strain curves was proposed by Belén et al. [21].

Nevertheless, research on the stress–strain relationship of carbonated RAC was rarely reported. This paper aims to investigate the influence of replacement ratio of RCA and carbonation degree on the mechanical properties of RAC. An analytical expression to describe the stress–strain curves of RAC after carbonation was established in the research.

## 2. Materials and Methods

### 2.1. Materials

#### 2.1.1. Cement

The cement applied in this test was 32.5 ordinary Portland cement manufactured by China Cement, Co., Ltd. (Nanjing, China). The physical and chemical characteristics of the cement are presented in Table 1.

#### 2.1.2. Coarse Aggregate

**Figure 1:**
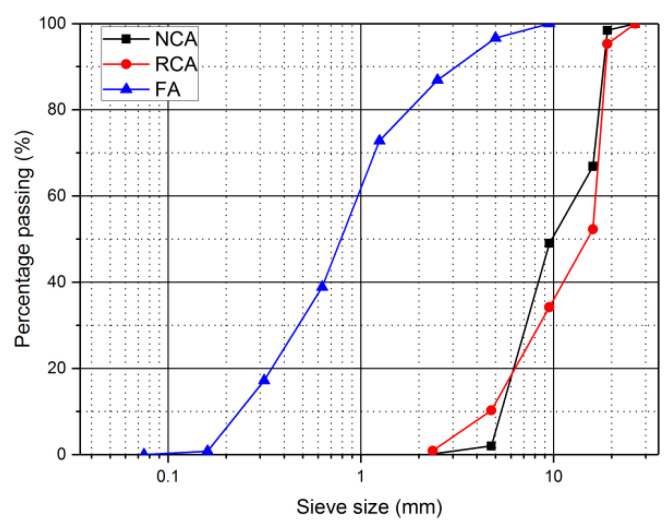
Grading distribution of natural coarse aggregate (NCA), recycled coarse aggregate (RCA) and fine aggregate FA.

The RCA provided by Nanjing Shoujia Renewable Resources Utilization Company (Nanjing, China) was made from waste concrete through mechanical crushing, cleaning, grading and processing. RCA and NCA were continuous-graded crushed gravel with a size from 2.5 to 26.5 mm. The basic properties of RCA and NCA tested according to JGJ 52-2006 [22] fulfilled the requirements. Figure 1 depicts the grading distributions of RCA and NCA, and Table 2 shows the basic properties of RCA and NCA.

#### 2.1.3. Fine Aggregate

Natural river sand with fineness modulus of 2.60 was selected as fine aggregate (FA). Figure 1 shows the grading distribution of FA, and Table 2 displays the basic properties of FA.

### 2.2. Mix Proportion

The mix proportion of the RAC was designed according to the method proposed in JGJ-55-2011 [23]. In order to achieve a target 28d cubic compressive strength (*f_cu,_*_28_) of 20 MPa for RAC with 100% RCA content, the water-cement ratio was determined to be 0.53 by tests. Additionally, considering the high water absorption characteristics of RCA, a suitable method should be sought to compensate the loss of water content, so as to reduce the influence of water loss on the mix design and improve the workability of RAC. Referring to the existing research literature, two effective methods were proposed to solve the aforementioned problem. One method was to add more water in the process of mixing the mixture [24,25], and the other method was to prewet the RCA with dry surfaces [26]. Referring to the research results of Poon et al. [27], Brand et al. [28] and Oliveira et al. [29], the RCA was soaked in water for 10 min, then taken out of the water and dried for 10 min before mixing. The concrete mixtures and properties are shown in Table 3.

### 2.3. Specimens

Four degrees of carbonation were designed including non-carbonation, partial carbonation with lower carbonation depth, partial carbonation with larger carbonated depth and full carbonation (non-carbonation represents a target carbonation depth of 0 mm; partial carbonation with lower carbonated depth represents a target carbonation depth of 15 mm; partial carbonation with larger carbonated depth represents a target carbonation depth of 35 mm; full carbonation represents a target carbonation depth of 50 mm).

Corresponding to each degree of carbonation, a total of four batches of concrete specimens were cast. Each batch consisted of six groups of concrete specimens with different replacement ratios of RCA (0%, 20%, 40%, 60%, 80% and 100%). Within each group, 3 cubic specimens (100 mm × 100 mm × 100 mm) were used to obtained the measured carbonation depth (*MD*), 3 cubic specimens were used to obtain the *f_cu,_*_28_, 6 cubic specimens were used to measure the cubic compressive strength after carbonation (*f_cu_*) and 6 prismatic specimens (100 mm × 100 mm × 300 mm) were applied for the tests of static elastic modulus (*E_c_*) and stress–strain curves after carbonation. A total of 288 cubic specimens and 144 prismatic specimens were prepared. All specimens were cured for 24 h, demolded, then covered with wet straw-matting for the first 7 days and placed in the laboratory until the 28th day.

### 2.4. Test Methods

#### 2.4.1. Accelerated Carbonation Test

Accelerated carbonation test was carried out in accordance with the method proposed in Chinese code GB/T 50082-2009 [30]. After 28 days of natural curing, the concrete specimens were placed in an air-dry oven for 48 h (60 °C). The concrete specimens were taken out of the oven, five sides of the specimen were sealed with heated paraffin, and the remaining unsealed side allowed the carbon dioxide to enter the inside of the specimen in one direction. Then the prepared concrete specimens were placed on the bracket of the HTX-12X carbonation chamber with CO_2_ concentration, relative humidity and temperature of (20 ± 2)%, (70 ± 5)% and (20 ± 5) °C, respectively. The distance between each concrete specimen was more than 50 mm.

The initial model for predicting the carbonation depth established based on the diffusion law shows that there is a good correlation between the carbonation rate and the carbonation duration [31]. A similar conclusion was also found by Neves et al. [32]. Therefore, the specimen could reach the expected carbonation depth by setting the predetermined carbonation duration. When the test carbonation duration approached the predetermined value, one cubic specimen was taken out every 15 d and split to test whether the carbonation depth reached the expected value.

#### 2.4.2. Carbonation Depth Measurement

**Figure 2:**
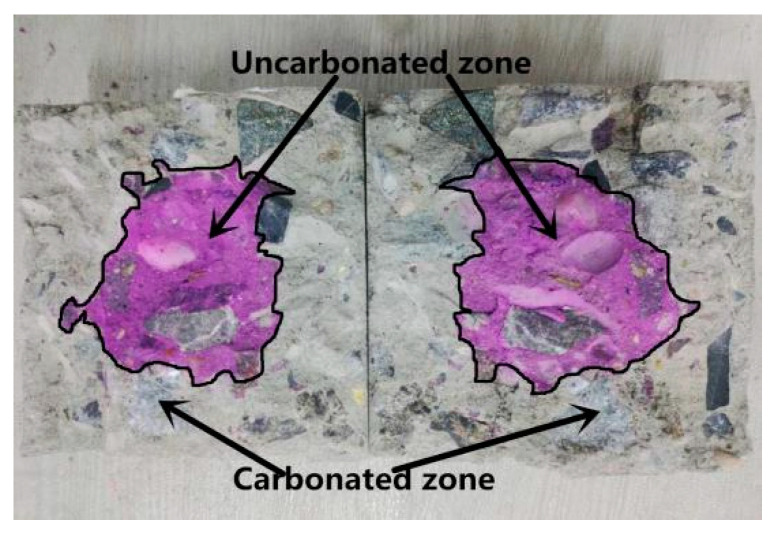
Cross section of the prepared specimen for measuring the carbonation depth.

As shown in Figure 2, the prepared cubic concrete specimens were split and the cross sections were sprayed with 1% phenolphthalein alcohol solution (Nanjing, China). After the color change was stable, the length from the edge of the specimen to the edge of the red-purple area was measured at intervals of 10 mm along the edge of the specimen. The mean value of the measured results was taken as the original carbonation depth. The average value of the carbonation depth of three identical samples was taken as the final measurement result. Table 4 lists the carbonation depth values of each batch of concrete specimens.

Carbonation depth was used as a single index to measure the degree of carbonation of concrete. For concrete structures or components with different cross-section dimensions, their mechanical properties were different to a certain extent although the measured carbonation depth values were the same. Therefore, the size of concrete structure and component should be considered when measuring the degree of concrete carbonation. Considering the size effect and more reasonably describing the change of mechanical properties of concrete after carbonation, the relative carbonation depth (*D*) of concrete, which is the ratio of the *MD* to half of the section side length of the cubic specimen, was defined as the analytical parameter to study the performance of carbonated concrete. The experimental results of the *D* of concrete specimens are listed in Table 4.

#### 2.4.3. Uniaxial Compressive Loading Test

After the carbonation test, the uniaxial compressive loading test was carried out. In order to avoid the friction force produced in the process of top-load application from affecting the stress state of the entire concrete specimen [33], two displacement sensors with a range of 200 mm were installed to measure the strain in the midspan of the specimen under uniaxial compression. In addition, two strain gauges were placed in the midspan of the other two sides of the specimen to measure transverse strain. A load sensor was placed between the bottom of the specimen and the loading plate to measure the axial load during the loading process. The digital display pressure testing machine with the capacity of 1000 kN was used to apply uniaxial compressive force to the specimen at the loading rate of 1.0 kN/s. The test data, which consisted of the longitudinal strain, the transverse strain and the axial load on the specimens, was collected by the dynamic signal acquisition and analysis system (20 points per second).

## 3. Results and Discussion

### 3.1. 28d Cubic Compressive Strength

**Figure 3:**
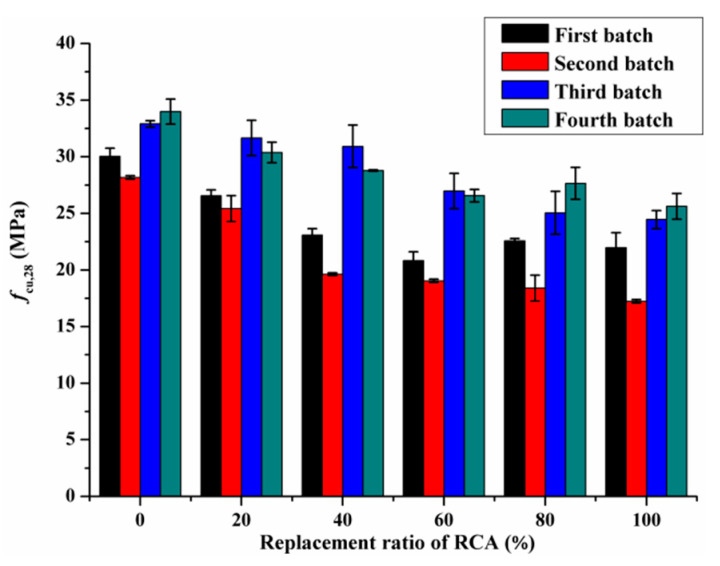
The *f_cu,_*_28_ of specimens versus the replacement ratio of RCA.

Figure 3 shows the *f_cu,_*_28_ of the RAC in dependence on the replacement ratio of RCA. It can be clearly seen that the *f_cu,_*_28_ of RAC presents a downward trend as the content of RCA increased. Compared with NAC, the *f_cu,_*_28_ of RAC with replacement ratio of 20%, 40%, 60%, 80% and 100% decreased by 8.9%, 18.2%, 25.6%, 25.2% and 28.6%, respectively. Similar experimental results were also reported by Akbarnezhad et al. [34].

The reduction of the *f_cu,_*_28_ of RAC due to the incorporation of RCA was mainly attributed to the following three aspects. Firstly, the bond between RCA and old and new mortar is weak, which is not conducive to the development of bond strength. Secondly, at the initial stage of mixing concrete, the excess water contained in RCA was released during the hydration process, leading to an increase in water-cement ratio, thus reducing the *f_cu,_*_28_ of RAC. Thirdly, RCA has large porosity, high crushing value and its strength is lower than NCA. With the increase of replacement ratio, stress concentration can easily occur in the process of axial pressure loading due to the accumulated damage existing in RCA.

**Figure 4:**
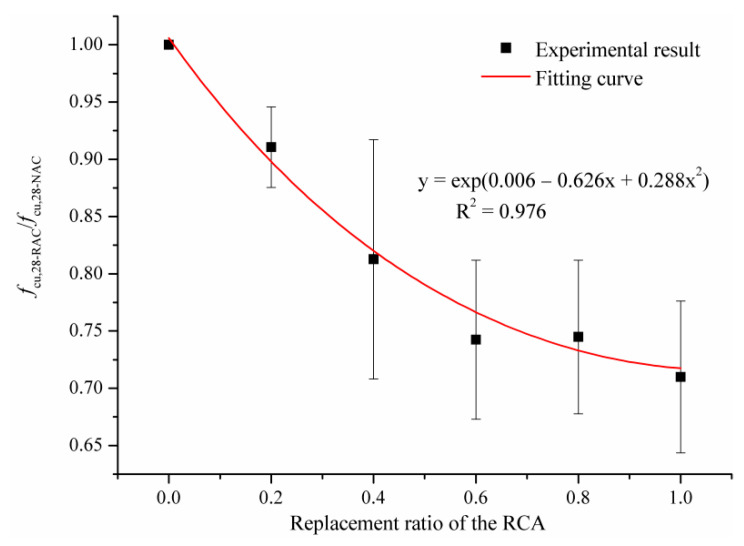
The relative *f_cu,_*_28_ in dependence on the replacement ratio of RCA.

**Figure 5:**
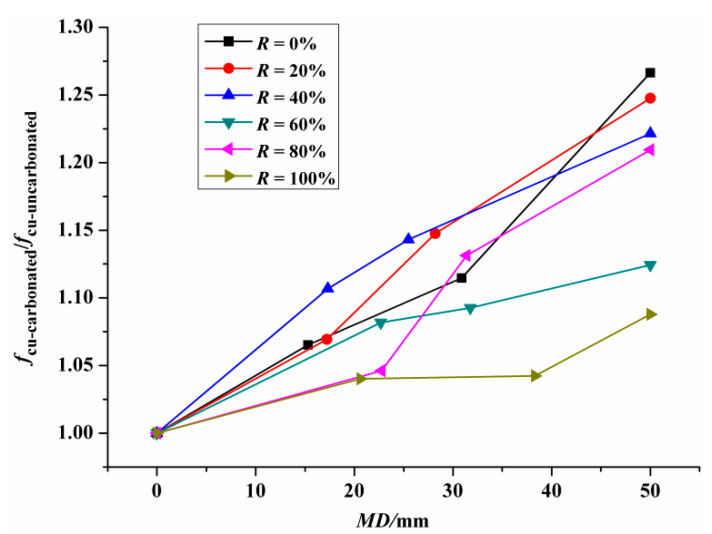
Relative cubic compressive strength of RAC versus the *MD.*

As analyzed by Hu et al. [35], due to the randomness of the experiment and the complex interaction mechanism between RCA and NCA, there is no monotonic relationship between *f*_cu,28_ and RCA substitution percentage. In this study, the ratio of the 28d cubic compressive strength of RAC (*f_cu,_*_28*-RAC*_) to the 28d cubic compressive strength of NAC (*f_cu,_*_28*-NAC*_) was defined as the relative *f_cu,_*_28_. Figure 4 shows the relationship between relative *f_cu,_*_28_ and RCA substitution percentage. The correlation coefficient (R^2^) of the fitted curve is higher than 0.9, indicating that the formula shown in Figure 4 could quantitatively measure the loss of *f_cu,_*_28_ at each RCA substitution rate. As can be observed from Figure 5, the decrease in relative *f_cu,_*_28_ with the replacement ratio ranging from 0% to 60% was significantly greater than that of the replacement ratio varying from 60% to 100%. This illustrates that the low content of RCA has a great impact on the *f_cu,_*_28*-RAC*_. This is closely related to the development path and process of internal cracks. In the process of exerting uniaxial compressive force, the initial cracks inside RAC specimens expanded, widened, and gradually penetrated the RCA and weak zones.

### 3.2. Cubic Compressive Strength after Carbonation

In this paper, under the same RCA substitution ratio, the ratio of the cubic compressive strength of carbonated concrete (*f_cu-carbonated_*) to that of uncarbonated concrete (*f_cu-uncarbonated_*) was defined as the relative cubic compressive strength after carbonation. Figure 5 shows the relative cubic compressive strength of RAC after carbonation in dependence on the *MD* at each specific RCA replacement ratio. As shown in Figure 5, at each specific substitution ratio, the relative cubic compressive strength of the RAC after carbonation presents an increasing trend with the increase of the *MD*. In terms of the RAC with 40% replacement ratio, the *f_cu_* of RAC with the carbonation depth of 17.3 mm, 25.5 mm and 50 mm were 10.7%, 14.3% and 22.2% higher than that of uncarbonated RAC, respectively.

In the process of carbonation, CO_2_ in the form of gas phase and liquid phase entered the concrete specimen mainly through the pores and cracks. The insoluble substance, CaCO_3_, formed by the chemical reaction between Ca(OH)_2_ and CO_2_ deposited in the pore structure; on the one hand, they could refine the size of the pores, on the other hand, their presence helps to reduce the volume of pores in cement-based material. Besides, the porosity of the loose concrete in ITZ was higher than that of the cement matrix, which was more conducive to the transmission of CO_2_ in the ITZ. The greater degree of carbonation further increased the strength of the concrete in ITZ. Therefore, the carbonated RAC shows higher compressive strength than uncarbonated RAC.

**Figure 6:**
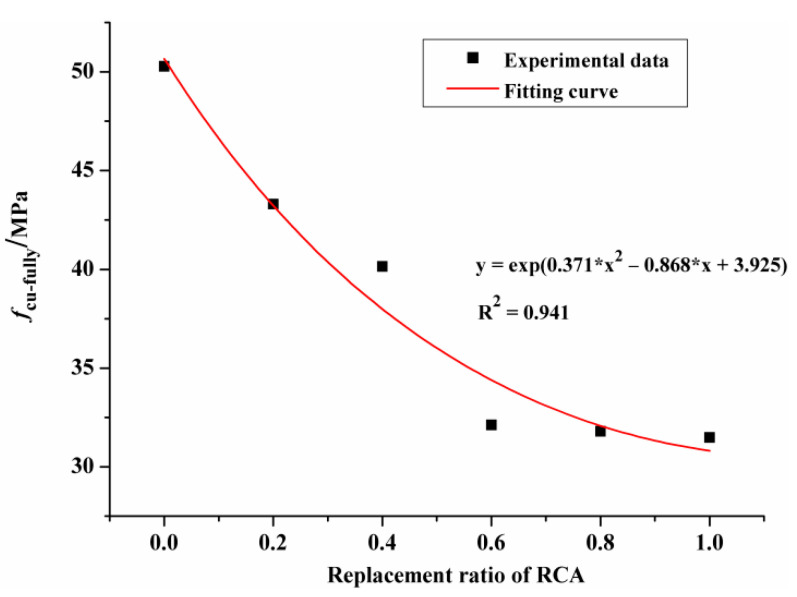
The *f_cu-fully_* versus the replacement ratio of RCA.

Figure 6 vividly depicts the relationship between the RCA substitution ratio and the cubic compressive strength of RAC after full carbonation (*f_cu-fully_*). As the content of RCA increased, the value of *f_cu-fully_* showed a gradual decline. This means that with the increase of RCA content, the *f_cu-fully_* was less affected by carbonation. This can be explained by the fact that most of the insoluble CaCO_3_ generated in the carbonation process was filled in the new mortar in RAC, and a very small part was filled in the old mortar and the interface between the new mortar and the old mortar. Compared with the new mortar, the bonding interface between the new and old mortar as well as the internal cracks of RAC were less affected by carbonation. Therefore, in a word, the defect of RCA was responsible for the strength loss of concrete specimens.

### 3.3. Static Elastic Modulus

Table 5 lists the test results of *E_c_* measured according to the method proposed in Chinese code GB/T 50082-2009 [30]. It can be seen that, the *E_c_* of NAC with relative carbonation depths of 0.31, 0.62 and 1 are 5.8%, 21.5% and 37.6% higher than that of uncarbonated NAC, respectively. For RAC with 100% replacement ratio, the *E_c_* of RAC with relative carbonation depths of 0.41, 0.77 and 1 are 4.9%, 9.6% and 13.5% higher than that of uncarbonated RAC, respectively. This is because the insoluble substance, CaCO_3_, generated in the process of carbonation constantly filled the pores of concrete, further improving the compactness of concrete, thus increasing the *E_c_* of the concrete.

**Figure 7:**
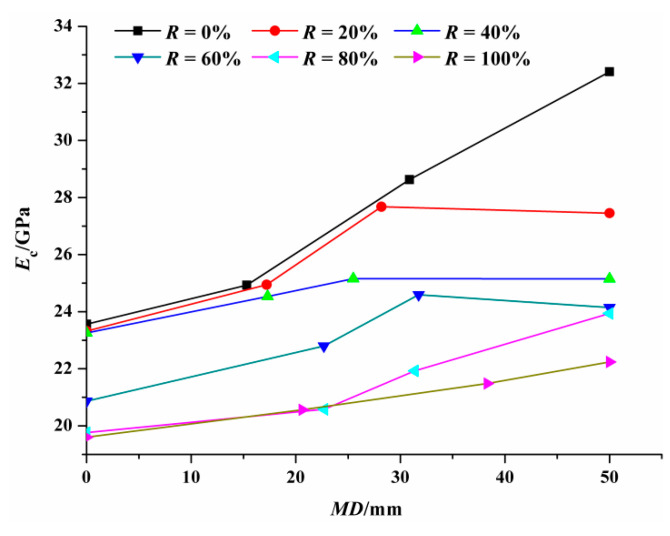
The *E_c_* of the RAC after carbonation versus the *MD*.

**Figure 8:**
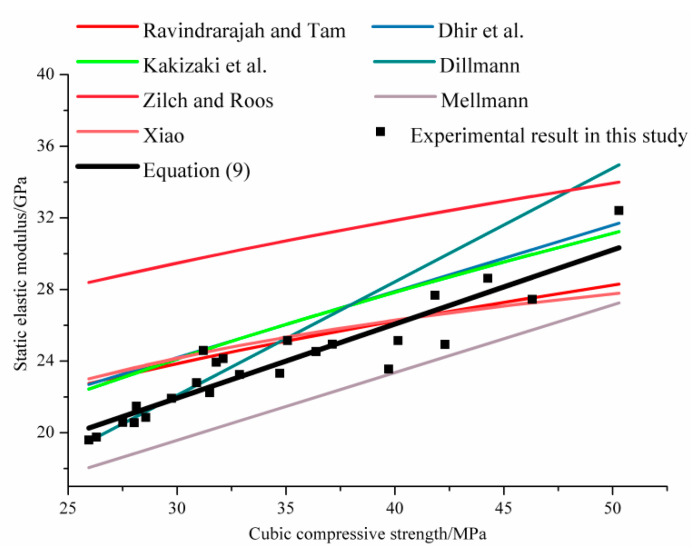
The static elastic modulus versus the cubic compressive strength of RAC.

Figure 7 shows the relationship between the *E_c_* and the *MD* of the carbonated RAC. It can be clearly observed that, under the condition of non-carbonation and complete carbonation, the *E_c_* of RAC with different replacement ratios was lower than that of NAC. Specifically, when the replacement ratio was 100%, the *E_c_* of uncarbonated RAC and fully carbonated RAC was 16.8% and 31.4% lower than that of NAC, respectively. Consistent research results were obtained by Chen et al. [36]. From Figure 8, it can also be concluded that greater replacement ratio of RCA led to smaller *E_c_* for RAC with the same carbonation degree. This is closely related to the porosity of the aggregate. The greater content of dense aggregate with high modulus of elasticity, the greater the *E_c_* of the concrete. The old mortar with high porosity attached to the surface of RCA and the original cracks inside RCA directly cause the elastic modulus of RCA to be lower than that of NAC.

Due to the different sources of RCA and the small number of test specimens, various forms of equations have been proposed by authors to characterize the relationship between the elastic modulus and the cubic compressive strength of carbonated RAC, as shown in Table 6. Based on the existing research results and the experimental results of this paper, the elastic modulus in dependence on the cubic compressive strength of carbonated RAC was depicted in Figure 8.

It can be seen intuitively that the existing proposed formulas cannot fit the experimental results well. In this paper, the alternative regression analysis was performed based on Equation (2), Equation (4) and Equation (6). The regression equation was as follows.

(8) $$E_{c}=a\cdot f_{cu}+b$$

where the regression coefficients *a* and *b* are 0.414 and 9.516, respectively. The correlation coefficient is R^2^ = 0.93. Thus, the following relationship was recommended to reckon the relationship between the static elastic modulus and the cubic compressive strength of carbonated RAC.

(9) $$E_{c}=0.414\cdot f_{cu}+9.516$$

### 3.4. Failure Pattern

**Figure 9:**
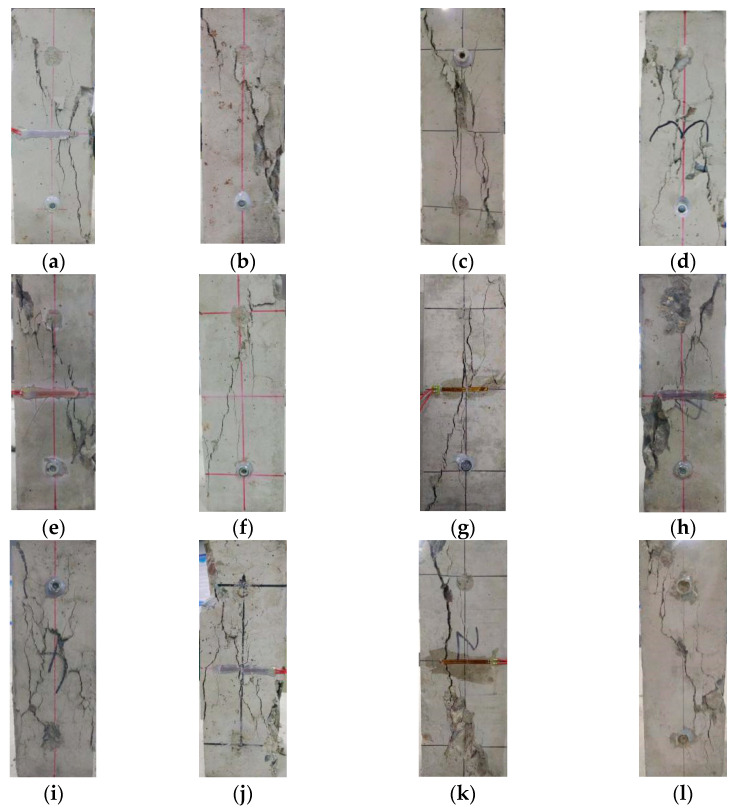
Failure patterns of NAC and RAC specimens after carbonation: (**a**) NC-1, (**b**) NC-2, (**c**) NC-3, (**d**) NC-4, (**e**) RC60-1, (**f**) RC60-2, (**g**) RC60-3, (**h**) RC60-4, (**i**) RC100-1, (**j**) RC100-2, (**k**) RC100-3 and (**l**) RC100-4.

Figure 9 shows the failure patterns of carbonated concrete specimens subjected to uniaxial compression load. With the increase of axial load, the carbonated NAC and RAC showed a similar failure process. When the applied stress exceeded the peak stress, tiny cracks parallel to the axial pressure appeared on the surface of the specimen. As the axial strain increased, these fine cracks gradually extended, widened and interconnected with each other. At last, an obvious oblique crack appeared on the surface of the specimen when failure occurred.

Compared with NAC, RAC specimens with high replacement ratios presented serious failure mode. Furthermore, the broken old and new mortar as well as RCA can be clearly seen on the fracture surface of the damaged RAC specimens. With respect to the failure process of the RAC with high substitution ratio, concrete specimens show brittle failure, marked by the rapid development of cracks and loss of bearing capacity. As can be seen in Figure 9e–h, compared with uncarbonated RAC, the surface spalling of concrete specimens with larger carbonation depth was observed, which was accompanied by a loud splitting sound in test, indicating that the carbonated RAC exhibits brittle characteristics. In general, failure planes observed from failed specimens indicate that the quality of the RCA is poor.

### 3.5. Uniaxial Compression Stress–Strain Curves

#### 3.5.1. The Influence of Carbonation on Stress–Strain Curves of RAC

**Figure 10:**
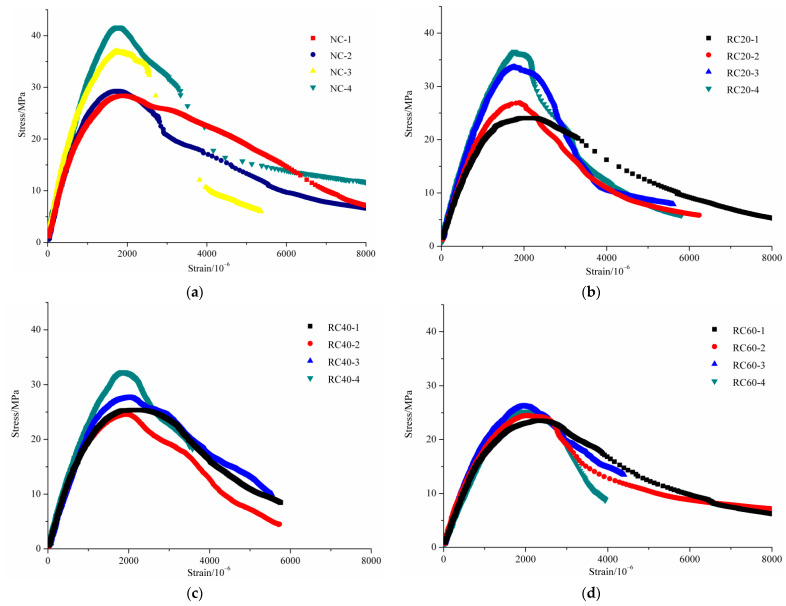

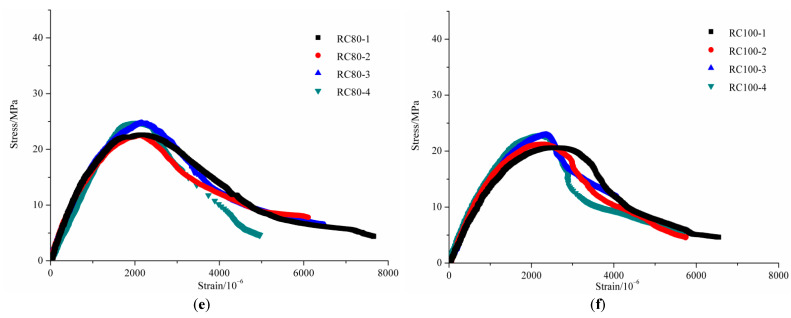
Stress–strain curves for NAC and RAC: (**a**) NAC, (**b**) RC20, (**c**) RC40, (**d**) RC60, (**e**) RC80 and (**f**) RC100.

Figure 10 shows the stress–strain curves of the carbonated concrete specimens under uniaxial compression. With the increase of vertical deformation, the stress of NAC and RAC with different carbonation depths shows a trend of first increasing and then decreasing. The peak stress (*σ_p_*) of NAC with relative carbonation depths of 0.31, 0.62 and 1 were 10.2%, 30.5% and 46.8% higher than that of uncarbonated concrete, respectively, and the corresponding peak strains (*ε_p_*) were 7.3%, 9.2% and 7.5% lower than that of uncarbonated concrete, respectively. Similar phenomenon could be seen from the comparison between the stress–stain curves of RAC with different carbonation depths in each figure. Furthermore, the greater the carbonation depth, the steeper the descending section of the stress–strain curve. This indicates that the carbonation would increase the brittleness of NAC and RAC. The reason for this was that the insoluble substances generated by carbonation gradually filled the pores and the cracks inside the concrete. With the increasing carbonation degree, the internal density of the concrete increased, resulting in a gradual increase in the ability of the entire specimen to bear external load, but a decrease in the ability to resist deformation.

It can be observed from Figure 10b–f that, with regard to the stress–strain curves of RAC with various carbonation depths, the difference was gradually reduced with the increasing replacement ratio. This illustrates that the stress–strain curves of RAC was less affected by carbonation with the increase of replacement ratio. This can be explained by the fact that the aforementioned insoluble substances produced during the carbonation process continuously filled the voids inside the mortar and the voids at the ITZ. Although the strength of concrete can be improved to a certain extent, the strength of RCA and the existence of original cracks inside RAC were not influenced by the carbonation process. Under the action of the external load, new cracks in RAC would continue to expand on the basis of these original cracks. Therefore, the original cracks existing in the interior of original natural aggregate inside RCA are the main cause of failure of RAC after carbonation.

#### 3.5.2. The Influence of Replacement Ratio on Stress–Strain Curves of RAC

**Figure 11:**
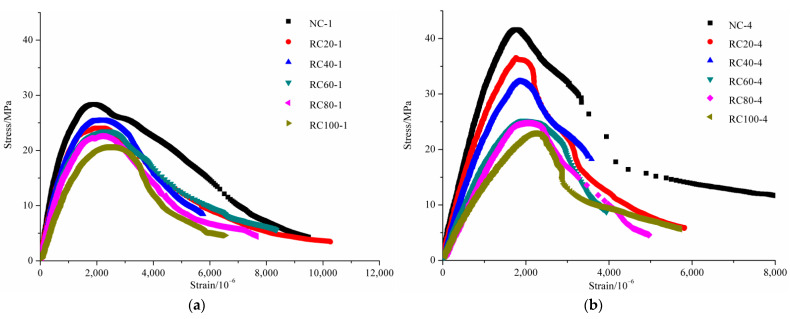
Stress–strain curves for RAC with the same carbonation depth: (**a**) uncarbonated concrete, (**b**) fully carbonated concrete.

Figure 11 shows the stress–strain curves of uncarbonated concrete and fully carbonated concrete with different substitution ratios. As can be seen from the stress–strain curve of the uncarbonated concrete shown in Figure 11a, the *σ_p_* decreased and the corresponding *ε_p_* increased with the increase of replacement ratio. The *σ_p_* of RAC with replacement ratios of 20%, 40%, 60%, 80% and 100% were 15.2%, 10.6%, 17.1%, 20.3% and 27.4% lower than that of NAC, respectively, and the corresponding *ε_p_* of RAC with replacement ratios of 20%, 40%, 60%, 80% and 100% were −0.8%, 3.8%, 9.4%, 16.8% and 25.6% higher than that of NAC, respectively. Suryawanshi et al. [18], Xiao et al. [20], Belén et al. [21] and Luo et al. [44] revealed a similar conclusion that the *σ_p_* of uncarbonated RAC was no more than 30% lower than that of NAC, and the *ε_p_* of RAC was no more than 25% higher than that of NAC. It was because with increasing content of RCA, in addition to the rising number of cracks inside mortar and ITZ, the effective water-cement ratio around RCA was larger than that around NCA due to the adopted prewetting method before mixing, thus leading to a decrease in *σ_p_*. With the increase of replacement ratio, the increase in *ε_p_* was attributed to two aspects. Firstly, under the action of external load, the progressive development of origin micro-cracks in RCA results in a tendency to increase the longitudinal strain at a faster rate than the applied stress. Additionally, compared with NCA, the larger water-cement ratio around RCA makes the RCA smoother, allowing the increase of strain in the ascending branch.

Figure 11b shows the stress–strain curves of fully carbonated concrete. A conclusion similar to the test results of uncarbonated concrete was obtained. For example, on the basis of the data analysis in Table 5, it can be found that the *σ_p_* of RAC with replacement ratios of 20%, 40%, 60%, 80% and 100% were 12.8%, 22.4%, 39.4%, 40.5% and 42.6% lower than that of NAC, respectively, and the corresponding *ε_p_* of RAC were 2.4%, 12.4%, 19.9%, 14.1% and 34.9% higher than that of NAC, respectively. Meanwhile, experimental results also illustrate that although the interior of the fully carbonated concrete has a high degree of compactness, a large amount of original cracks inside RCA was responsible for the failure of carbonated RAC. In addition, under the premise of the same degree of carbonation, the descending of the stress–strain curves of RAC gradually became steeper with the increasing replacement ratios of RCA, indicating that the brittleness of RAC increases due to the defect of RCA.

## 4. Fitting Analysis of Stress–Strain Relation of RAC after Carbonation

In previous studies, researchers were devoted to the investigation on the stress–strain relationship of RAC and proposed corresponding models [18,20,21]. The model proposed by Guo [45] was used to describe the stress–strain curves of RAC after carbonation. The normalized equation is shown as follows:

(10) y={ax+(3−2a)x2+(a−2)x3 0<x≤1xb(x−1)2+x                    x>1

(11) $$x=\frac{\varepsilon }{\varepsilon_{p}},y=\frac{\sigma }{\sigma_{p}},$$

where *a* and *b* denote the shape parameters of the ascending branch and descending branch of curves, respectively.

Combined with the experimental results and applying Equations (10) and (11), the optimal values of shape parameters *a*, *b* and the corresponding correlation coefficient (R^2^) were obtained through data regression analysis, which were all listed in Table 7, respectively. As shown in Table 7, the corresponding correlation coefficients are greater than 0.9, demonstrating that the application of the model proposed by Guo [45] could better describe the stress–strain relationship of RAC after carbonation.

**Figure 12:**
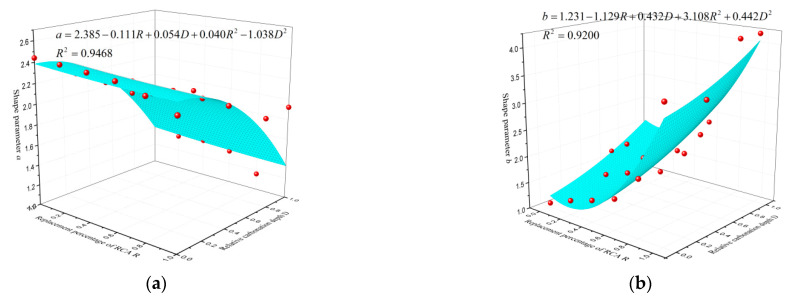
Three-dimensional fitting of shape parameters: (**a**) shape parameter *a*, (**b**) shape parameter *b.*

To investigate the influence of the two factors of replacement ratio and carbonation degree on the stress–strain relationship of RAC, the parameters *a*, *b* in dependence on the replacement ratio (*R*) and the value *D* are depicted in Figure 12, respectively. The fitting equations are shown as follows:

(12) a=2.385−0.111R+0.054D+0.040R2−1.038D2  R2=0.9468

(13) b=1.267−1.740R−0.237D+3.831R2+1.139D2  R2=0.9086

As shown in Figure 12a, the value of parameter *a*, which represents the slope of the initial tangent of the stress–strain curve, decreased gradually with the increase of replacement ratio and carbonation degree, demonstrating that the plastic deformation characteristic of RAC specimens is more obvious. As for parameter *b* shown in Figure 12b, it shows an increasing trend with the increasing replacement ratio and carbonation degree, indicating that the RAC specimen exhibited poor ductility [44]. However, some data points are not in good agreement with the fitting results. The reason for this was that the cracks inside specimens and microcracks in RCA developed randomly when the stress–strain curve entered the descending phase, which significantly affected the deformation of the concrete. In addition, this was explained clearly by Xiao et al. [46], that the stress–strain relationship of RAC, which consists of the ascending and descending branch, had larger variability than that of NAC.

Experimental results show that replacing NCA with RCA in concrete mix and conducting carbonation would significantly increase the brittleness of RAC. With regard to the increase of replacement ratio of RCA increasing the brittleness of RAC, it was because the RCA particles are softer than the NCA particles, the path of crack development was to penetrate the interior of the aggregate instead of extending along the periphery of the aggregate, which results in a reduction in fracture energy and ductility. Regarding the carbonation behavior increasing the brittleness of RAC, it may be related to the product of hydration reaction. In the process of hydration reaction, a large amount of Ca(OH)_2_ crystal, 3CaO·2SiO_2_·3H_2_O and 2CaO·SiO_2_·4H_2_O cementitious material with good deformation performance was generated inside the concrete. However, these crystals and cementitious materials would be consumed during the process of carbonation. In the absence of external force, the CaCO_3_ precipitates on the pore wall due to the reaction, further reducing the deformation performance of concrete.

**Figure 13:**
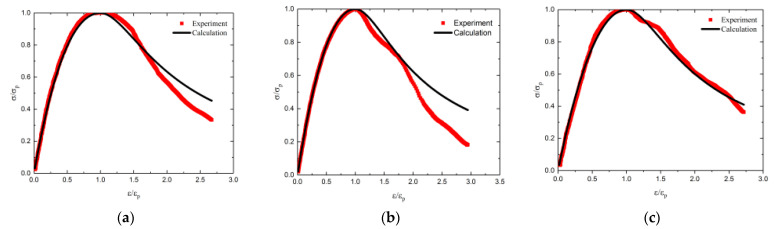

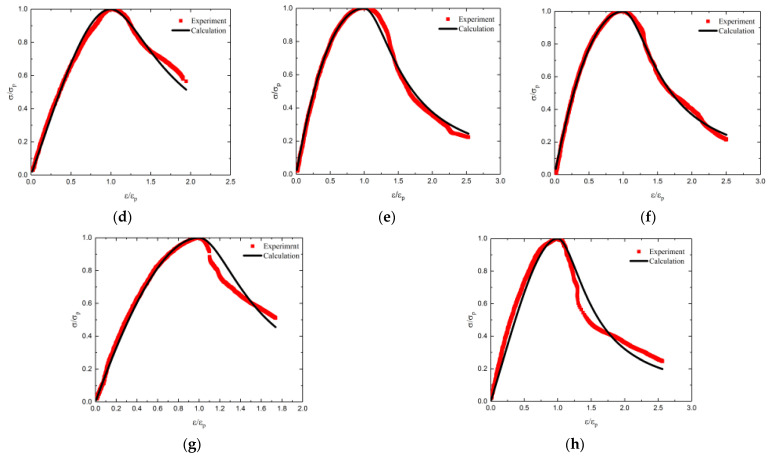
The comparison between the calculated and experimental stress–strain curves for RC40 and RC100: (**a**) RC40-1, (**b**) RC40-2, (**c**) RC40-3, (**d**) RC40-4, (**e**) RC100-1, (**f**) RC100-2, (**g**) RC100-3 and (**h**) RC100-4.

Figure 13 shows the comparison between the calculated dimensionless stress–strain curves and the experimental stress–strain curves for RAC with 40% and 100% substitution ratio at different carbonation depths. There is a high degree of agreement between the experimental curves and the calculated curves. Therefore, the stress–strain model put forward in this paper could be adopted to obtain the dimensionless stress–strain curves of RAC after carbonation.

## 5. Conclusions and Prospectives

With regard to the 28d cubic compressive strength of RAC with replacement ratio less than 60%, it decreased sharply with the increasing replacement ratio. When the content of RCA was larger than 60%, the 28d cubic compressive strength of RAC showed a slow decline with the replacement ratio. With regard to the NAC or RAC at a specific replacement ratio, greater degree of carbonation could further increase the cubic compressive strength.

The static elastic modulus of carbonated RAC gradually increased with the increasing carbonation depth. For uncarbonated or fully carbonated RAC, the static elastic modulus decreased with the increase of RCA content. The model for the relationship between static elastic modulus and cubic compressive strength was established according to the experimental results in this study.

Compared with the stress–strain curve of uncarbonated concrete, the influence of carbonation on the stress–strain curve of carbonated concrete was mainly reflected in the increase of stress value and the decrease of strain value. For RAC with high content of RCA, the stress–strain curves of RAC with various carbonation depth exhibited a small difference, indicating that the carbonation caused a lower impact. Under the condition of the same carbonation degree, the influence of RCA replacement ratio on the stress–strain curves was mainly reflected in the decrease of peak stress and the increase of peak strain.

The comparison results of the calculated stress–strain curves and the experimental stress–strain curves illustrates that the application of the existing model could better describe the stress–strain relationship of RAC after carbonation.

The mathematical model of the stress–strain curve for carbonated RAC proposed in this paper was established based on the existing test data. Reliability analysis should be added to further research if this model was applied to actual engineering. Additionally, the water-cement ratio of RAC was determined based on the adaptation of C20 strength concrete, and its applicability was limited. Further study on the mechanical properties of recycled high-strength concrete after carbonation is necessary.

## Figures and Tables

**Table 1:** Characteristics of the cement.

Characteristics	Value
Type	P.O. 32.5
Specific surface area (m^2^/kg)	367
Average diameter (µm)	15
Initial and final setting times (min)	163/223
Degree of fineness (%)	2.8
Loss on ignition (%)	4.16
3-day compressive and flexural strength (MPa)	16.5/4.0
Silicon oxide (SiO) (% by mass)	22.25
Aluminum oxide (Al_2_O_3_)	6.11
Ferric oxide (Fe_2_O_3_)	3.25
Calcium oxide (CaO)	59.43
Magnesium oxide (MgO)	1.42
Sulphate oxide (SO_3_)	3.31
Potassium oxide (K_2_O)	1.08
Sodium oxide (Na_2_O)	0.43

Note: P.O.—the code of ordinary portland cement.

**Table 2:** The basic properties of RCA, NCA and FA.

Property	Type		
	RCA	NCA	FA
Apparent density (kg/m^3^)	2356	2570	2629
Bulk density (kg/m^3^)	1286	1330	1666
Crushing value (%)	16.1	9.96	-
Mud content (%)	3.03	0.35	1.7
Water absorption (%)	4.1	0.76	-
Grain grading	II	II	II

**Table 3:** Design mixture proportions of concrete.

Property	Type					
	NC	RC20	RC40	RC60	RC80	RC100
Replacement ratio of RCA (%)	0	20	40	60	80	100
Water-cement ratio	0.53	0.53	0.53	0.53	0.53	0.53
Cement (kg/m^3^)	368	368	368	368	368	368
Fine aggregate (kg/m^3^)	619	619	619	619	619	619
NCA in dry state (kg/m^3^)	1050	840	630	420	210	0
RCA in dry state (kg/m^3^)	0	210	420	630	840	1050
Water (kg/m^3^)	195	195	195	195	195	195

**Table 4:** Carbonation test results of concrete specimens.

Degree of Carbonation	Property	Type					
		NC	RC20	RC40	RC60	RC80	RC100
non-carbonation	*MD* (mm)	0	0	0	0	0	0
	*D*	0	0	0	0	0	0
	*S* (mm)	0	0	0	0	0	0
	*T* (days)	0	0	0	0	0	0
partial carbonation	*MD* (mm)	15.3	17.2	17.3	22.7	22.7	20.6
	*D*	0.31	0.34	0.35	0.45	0.45	0.41
	*S* (mm)	3.47	3.14	2.34	3.05	3.19	3.35
	*T* (days)	75	75	75	75	75	60
partial carbonation	*MD* (mm)	30.9	28.2	25.5	31.8	31.4	38.3
	*D*	0.62	0.56	0.51	0.64	0.63	0.77
	*S* (mm)	2.45	3.52	2.67	2.79	3.09	2.98
	*T* (days)	165	120	105	120	120	120
full carbonation	*MD* (mm)	50	50	50	50	50	50
	*D*	1	1	1	1	1	1
	*S* (mm)	0	0	0	0	0	0
	*T* (days)	210	210	210	210	210	210

Note: *S*—the standard deviation for mean values among cubic concrete specimens; *T*—the time which concrete specimens were exposed to carbonation; *MD*—measured carbonation depth; *D*—the relative carbonation depth.

**Table 5:** Properties of RAC after carbonation.

Type	Property				
	Replacement Percentage of RCA (%)	Relative Carbonation Depth *D* (mm)	Static Elastic Modulus *E_c_* (GPa)	Peak Stress *σ_p_* (MPa)	Peak Strain *ɛ_p_* (10^−6^)
NC-1	0	0	23.56	28.36	1917.72
NC-2		0.31	24.93	31.24	1778.24
NC-3		0.62	28.63	37.01	1741.83
NC-4		1	32.41	41.62	1773.21
RC20-1	20	0	23.32	24.03	1963.08
RC20-2		0.34	24.95	26.95	1886.97
RC20-3		0.56	27.69	33.65	1772.42
RC20-4		1	27.45	36.55	1758.85
RC40-1	40	0	23.25	25.35	2155.28
RC40-2		0.35	24.53	24.61	1945.22
RC40-3		0.51	25.15	27.58	2040.34
RC40-4		1	25.15	32.31	1841.14
RC60-1	60	0	20.86	23.50	2298.69
RC60-2		0.45	22.80	24.46	2044.33
RC60-3		0.64	24.59	26.15	2016.69
RC60-4		1	24.15	25.22	1939.13
RC80-1	80	0	19.76	22.60	2187.25
RC80-2		0.45	20.58	22.55	2115.91
RC80-3		0.63	21.93	24.71	2166.39
RC80-4		1	23.94	24.78	2071.06
RC100-1	100	0	19.60	20.58	2587.14
RC100-2		0.41	20.56	21.21	2298.69
RC100-3		0.77	21.48	22.89	2362.11
RC100-4		1	22.24	23.90	2227.97

**Table 6:** Equations that correlate elastic modulus with cubic compressive strength of RAC.

Author	Equation	
Ravindrarajah et al. [37]	$E_{c}=7.770{f_{cu}}^{0.33}$	(1)
Dhir et al. [38]	$E_{c}=0.370f_{cu}+13.100$	(2)
Kakizaki et al. [39]	$E_{c}=190\times {\left(\frac{\rho }{2300}\right)}^{1.5}\times \sqrt{\frac{f_{cu}}{2000}}$	(3)
Dillmann [40]	$E_{c}=0.63443f_{cu}+3.0576$	(4)
Zilch et al. [41]	$E_{c}=9.100\times {\left(f_{cu}+8\right)}^{1/3}\times {\left(\frac{\rho }{2400}\right)}^{2}$	(5)
Mellmann [42]	$E_{c}=0.378f_{cu}+8.242$	(6)
Xiao et al. [43]	$E_{c}=\frac{10^{2}}{2.2+\frac{40.1}{f_{cu}}}$	(7)

**Table 7:** The shape parameters *a*, *b* and corresponding correlation coefficient.

Type	Property				
	*D*	*a*	Correlation Coefficient (R^2^)	*b*	Correlation Coefficient (R^2^)
NC-1	0	2.44	0.9989	0.685	0.929
NC-2	0.31	2.204	0.9986	1.214	0.9982
NC-3	0.62	2.064	0.9989	2.185	0.9772
NC-4	1	1.475	0.9986	1.051	0.9948
RC20-1	0	2.420	0.9989	1.27	0.9833
RC20-2	0.34	2.157	0.9992	2.416	0.9986
RC20-3	0.56	1.981	0.999	2.374	0.9766
RC20-4	1	1.357	0.9997	2.887	0.998
RC40-1	0	2.439	0.9989	1.513	0.9645
RC40-2	0.35	2.222	0.9993	1.971	0.9694
RC40-3	0.51	2.051	0.9995	1.25	0.989
RC40-4	1	1.378	0.9993	1.777	0.9865
RC60-1	0	2.378	0.9993	1.478	0.9978
RC60-2	0.45	2.153	0.9993	1.141	0.9916
RC60-3	0.64	2.094	0.9994	1.622	0.9938
RC60-4	1	1.334	0.999	2.361	0.9566
RC80-1	0	2.307	0.9986	1.971	0.9933
RC80-2	0.45	2.135	0.9993	2.003	0.9892
RC80-3	0.63	2.006	0.9992	2.343	0.9978
RC80-4	1	1.164	0.9995	4.041	0.996
RC100-1	0	2.204	0.9985	3.470	0.9846
RC100-2	0.41	2.14	0.9948	3.291	0.9953
RC100-3	0.77	1.896	0.9991	4.196	0.9252
RC100-4	1	1.924	0.9991	4.208	0.9836

## Data Availability

The data presented in this study are available on request from the correspondence author.
